# Five Weeks of Whole-body Vibration in Prehabilitation for Knee Function Following Anterior Cruciate Ligament Reconstruction: A Single-blinded Randomized Controlled Trial

**DOI:** 10.1186/s40798-025-00901-1

**Published:** 2025-08-22

**Authors:** Jihong Qiu, Michael Tim-Yun Ong, Chi-Yin Choi, Mingde Cao, Violet Man-Chi Ko, Xin He, Sai-Chuen Fu, Daniel T.P. Fong, Patrick Shu-Hang Yung

**Affiliations:** 1 School of Exercise and Health, Shanghai University of Sport, Shanghai, China; 2 Department of Orthopaedics and Traumatology, Faculty of Medicine, The Chinese University of Hong Kong, Shatin, Hong Kong SAR China; 3 National Centre for Sport and Exercise Medicine, School of Sport, Exercise and Health Sciences, Loughborough University, Loughborough, LE11 3TU UK; 4 Prince of Wales Hospital, Room 74029, 5F, Lui Che Woo, Clinical Science Building, Shatin, NT, Hong Kong SAR China

**Keywords:** Whole-body vibration, Knee extensors, Physical therapy

## Abstract

**Background:**

Good preoperative quadriceps neuromuscular function is associated with satisfactory functional outcomes post-anterior cruciate ligament reconstruction (ACLR). Whole-body vibration (WBV), which can modulate quadriceps neuromuscular function has not yet been incorporated into ACL prehabilitation. The aim of this study was to determine whether the combination of WBV in a prehabilitation program could achieve a better knee function after ACLR by promoting quadriceps neuromuscular function during the preoperative period.

**Methods:**

A single-blinded randomized controlled clinical trial was conducted. Forty-four participants with a primary, unilateral ACL rupture were randomly assigned to the control (*N* = 22) or the WBV group (*N* = 22). The control group underwent prehabilitation twice weekly for five weeks. The WBV group received the same prehabilitation plus WBV. Quadriceps neuromuscular function, including strength (maximal voluntary isometric contraction, MVIC), the early (rate of torque development from 0 to 50ms, RTD_0 − 50_), and the late phase (rate of torque development from 100 to 200ms, RTD_100 − 200_) of rapid contraction and inhibition (central activation ratio, CAR) in the injured limb, were measured at baseline and preoperatively. Knee function was assessed by the International Knee Documentation Committee (IKDC) Score at baseline, preoperatively, and 4 months postoperatively. The linear mixed effect models and multiple linear regression were used for the statistical analyses.

**Results:**

Forty participants completed interventions and 35 finished the postoperative follow-up. Preoperatively, the intervention demonstrated main effects on quadriceps MVIC (*p* = 0.002), RTD_0 − 50_ (*p* = 0.024), RTD_100 − 200_ (*p* = 0.005) and CAR (*p* = 0.043). Furthermore, the effect of time* intervention interaction was significant on quadriceps MVIC (*p* = 0.011). Postoperatively, the WBV group achieved higher IKDC scores than the control group (*p* = 0.006). The improvements in preoperative quadriceps MVIC and intervention contributed to better knee function post-ACLR (R^2^ = 0.239, *p* = 0.007).

**Conclusions:**

Five weeks of WBV in prehabilitation enhanced quadriceps strength pre-ACLR and had potential to enhance knee function post-ACLR. WBV can be considered as an adjunct to prehabilitation protocols.

**Trial Registration:**

ClinicalTrials.gov, NCT04988828. Registered 3rd August 2021, https://clinicaltrials.gov/study/NCT04988828?cond=anterior%20cruciate%20ligament&term=WBV&locStr=Hong%20Kong&country=Hong%20Kong&rank=3.

**Supplementary Information:**

The online version contains supplementary material available at 10.1186/s40798-025-00901-1.

## Key Points

1. Patients receiving WBV during prehabilitation showed higher IKDC scores 4 months post-ACLR compared to standard prehabilitation alone.

2. Preoperative quadriceps strength improvements and the WBV therapy were positively associated with better knee function following ACL reconstruction, highlighting the importance of effective prehabilitation strategies.

3. WBV appears to be a valuable adjunct therapy for enhancing quadriceps function before ACL surgery, potentially establishing a stronger foundation for postoperative recovery.

## Background

Anterior cruciate ligament (ACL) injuries are prevalent in sports. To restore mechanical stability and facilitate return to sports, approximately 80% of patients opt for ACL reconstruction (ACLR) as their first-line treatment after injury [1]. Despite the success of ACLR and rehabilitation, patients frequently encounter persistent quadriceps weakness, suboptimal functional performance, and impaired knee function, hindering their ability to fully return to pre-injury levels of sports activity [2, 3]. Recognizing the challenges faced by individuals post-ACLR, experts in orthopedics reached a consensus at the Panther Symposium [4], recommending that patients shall undergo a period of progressive rehabilitation, commonly referred to as prehabilitation (Prehab) before the ACLR [4]. The consensus underscores the importance of Prehab in the continuum of care for ACL injuries, highlighting its potential to optimize patient outcomes and facilitate a successful return to sports.

Giesche et al. [5] summarized that several studies have explored the effects of Prehab on postoperative outcomes in individuals undergoing ACLR. Notably, no standardized protocol for Prehab before ACLR has been established [5]. Following the alleviation of pain, swelling, and range of motion deficits, these studies implemented diverse exercise modalities in their Prehab programs, including proprioceptive and balance training, along with strength and plyometric training targeting quadriceps, hamstring, and gluteus muscles [5]. Quadriceps strength, time from injury, patient-reported outcomes, and functional performance prior to ACLR have been identified as factors influencing postoperative quadriceps strength, patient-reported outcomes, and the likelihood of returning to sports [6–8]. Recent research also highlighted the significance of rapid quadriceps contraction and quadriceps inhibition before ACLR as predictors of postoperative outcomes [9, 10]. However, previous Prehab programs lacked specific interventions targeting rapid quadriceps contraction and inhibition. Consequently, this study aims to investigate whether incorporating such interventions into Prehab can enhance knee function after ACLR.

Whole-body vibration (WBV), characterized by vibratory stimuli emanating from a platform and transmitted throughout the entire body, has shown potential benefits such as increased quadriceps strength, improved rapid quadriceps contraction, and reduced quadriceps inhibition. Specifically, studies indicate that mechanical stimuli from WBV platforms can stimulate muscle anabolism, elevate the proportion of type II muscle fibers, enhance corticospinal excitability, and modulate the gamma system in the quadriceps [11–13]. These physiological determinants are integral to quadriceps strength, inhibition, and rapid contraction [14–16]. However, the effects of WBV on these quadriceps neuromuscular functions in patients with ACL deficiency remain unclear. In addition, the application of WBV in Prehab before ACLR has not been reported previously. Therefore, the main objective of this study was to determine whether the combination of WBV in a Prehab program could achieve a better knee function after ACLR by promoting neuromuscular function of the quadriceps muscle during the preoperative period. We hypothesized that patients undergoing Prehab with WBV could achieve better knee function post-ACLR. Additionally, patients with more improvements in the quadriceps neuromuscular functions prior to ACLR would achieve better knee function following the surgery.

## Methods

### Study Design

This study was a single-center, single-blinded, randomized controlled clinical trial conducted at the Prince of Wales Hospital, Shatin, Hong Kong following Consolidated Standards of Reporting Trials guidelines. The study was approved by the Joint Chinese University of Hong Kong-New Territories East Cluster Clinical Research Ethics Committee (No.2019.488) in October 2019, and the trial was registered with ClinicalTrials.gov (NCT04988828). The study was performed in accordance with the standards of ethics outlined in the Declaration of Helsinki.

### Participant Recruitment and Eligibility

Participants were consecutively enrolled in the study between August 2021 and July 2022. Participants were eligible if they (1) were aged 18–45 years, (2) had a body mass index (BMI) < 29 Kg/m^2^, (3) had a primary, unilateral ACL injury, (4) were engaged in sports regularly before the injury (pre-injury activity level ≥ Tegner Activity Scale of 6, ≥twice/week), (5) exhibited no pain, swelling, or range of motion deficits in the knee joint at the study screening, (6) received and completed the standard Prehab, ACLR with the hamstring autograft, and the standard postoperative rehabilitation in our institution. While prior treatments or physiotherapy were not restricted, participants were required to adhere to the standard treatments provided by our institution upon consenting to participate in this study. Participants were excluded from this study if they (1) had undergone previous ACLR or other surgeries to either knee joint, (2) had sustained any injury to either lower extremity in the last 6 months, (3) had concomitant bone fractures, knee dislocations, or multi-ligamentous ruptures, (4) had any cardiovascular, metabolic, or neurological disorders, (5) failed to undergo the scheduled surgery at our institution, or quit/did not take the standard postoperative rehabilitation in our institution. Participants were not excluded based on concomitant bone bruises or meniscal injuries due to the high prevalence of these injuries in the population with ACL injury [17, 18]. However, concomitant meniscal injuries requiring extra operations during ACLR were documented. All participants gave written informed consent in accordance with the Declaration of Helsinki before their participation, and all procedures were adhered to the ethical guidelines.

An a priori power analysis conducted using G*Power 3.9 determined that 18 participants per group would provide 80% statistical power. Sample size estimation was based on a pilot study of 10 participants (5 per group) in this study. In this pilot, the WBV group showed a mean improvement in IKDC scores of 11.2 ± 4.3 from baseline to 4 months post-ACLR, while the control group showed an improvement of 6.5 ± 3.8, yielding an effect size of 0.59. The power calculation assumed α = 0.05, 2 groups, 3 repeated measurements, and a correlation of 0.5 among repeated measures. Considering an anticipated dropout rate of 20%, the final sample size was set at 22 participants per group.

### Randomization and Blinding

Eligible participants underwent random allocation to either the control or WBV group, facilitated by a 4-block randomization strategy through an online generator (http://www.randomization.com) before the baseline assessment. A research assistant was responsible for creating the randomization sheet and concealing the individual allocations within sealed envelopes. Both the participants and the investigators remained unaware of the group assignment until the initiation of their first Prehab session. Only the outcome assessor maintained blinding to the treatment allocation in the whole study process, ensuring impartial evaluation.

### Prehab and Intervention

All the participants received a standard Prehab program for ACLR administered by our institution, comprising twice-weekly sessions over a period of 5 weeks. All participants started their Prehab and the intervention 6–7 weeks before their scheduled surgery (control group: 6.4 ± 0.5 weeks, WBV group 6.3 ± 0.4 weeks, *p* = 0.43). Each session, guided by JH Qiu, encompassed a 5 min of warm-up, proprioceptive training, and a series of strengthening exercises targeting quadriceps, hamstring, and gluteus muscles. The detailed descriptions of the Prehab program were organized according to the Consensus on Exercise Reporting Templates (CERT) [19], which can be found in Supplementary File 1. Functional training, consisted of progressively challenging exercises including jogging, deep jump with soft landing, as well as forward, lateral and cross-over single leg hopping, was also integral to each Prehab session. The exercises progressed from double-legged maneuvers (e.g., jogging and deep jump with soft landing) to simple single legged maneuvers (e.g., forward and lateral single leg hop), and finally to complex single legged maneuvers (e.g., cross-over single leg hop). Regular evaluations of exercise progression occurred on a weekly basis, taking into account the individual clinical condition of each participant. Adjustments to the strengthening exercise intensity were made in accordance with the progression models recommended by the American College of Sports Medicine [20].

The WBV group received WBV treatment plus the standard Prehab. The participants performed 6 bouts of 60 s of squats with the knee flexed at approximately 60° on a vertical WBV platform with 1 min rest interval between each bout (PhysioPlate My Gold, Globus, Codognè, Italy) [21]. Knee flexion angle was controlled using a goniometer to position participants initially, followed by placement of an adjustable ruler at the height of the knee joint to provide reference for a physiotherapist. The physiotherapist visually monitored the knee height relative to the ruler to ensure maintenance of the proper knee angle throughout each bout. To well activate the quadriceps, the frequency of the WBV stimuli from the platform was 30 Hz, and the amplitude was 4 mm [22]. To ensure equivalence in the squat exercises for the control group, identical squats with the same protocol as the WBV group were performed on the floor. To mitigate the influence of squat exercises on quadriceps function, the control group’s protocol mirrored that of the WBV group. Considering the potential interactions between the intervention and the exercises in Prehab, the participants completed the warm-up and proprioception training first, then were exposed to WBV (or squat exercises), and then performed quadriceps strengthening exercises in each session [23].

### Surgical Techniques and Postoperative Rehabilitation

All surgical procedures in the study were performed by 3 experienced orthopaedics surgeons from the same team. The participants had a standard arthroscopic autologous hamstring reconstruction with the femoral and the tibial side fixed with an endo-button and interference screw, respectively. After surgery, participants were allowed to mobilize, and they commenced physiotherapy in the first week after discharge. Our institution’s standard postoperative rehabilitation regime had been reported previously [24].

### Outcome Measurements

Data collection in this study occurred at three distinct time points: baseline, preoperatively, and 4 months postoperatively. Our decision to limit the postoperative follow-up period to 4 months was primarily driven by the uniformity in postoperative rehabilitation during this period. Sport-specific exercises in rehabilitation for ACLR typically commences after 4 months postoperatively [25], which will confound the results of interest. Besides, the outcomes at 4 months after ACLR have been demonstrated to be indicative of long-term outcomes [26]. At baseline and preoperatively, maximal quadriceps strength, quadriceps rapid contraction capacity, quadriceps inhibition, and subjective knee function were assessed. At 4 months postoperatively, subjective knee function was measured again. The preoperative assessment took place within 1–2 weeks before the surgery (control group: 1.5 ± 0.4 weeks, WBV group: 1.4 ± 0.3 weeks, *p* = 0.37). For the postoperative assessment, participants completed the online IKDC questionnaire between the 16th and 18th weeks after surgery (control group: 16.8 ± 1.7 weeks, WBV group: 17.1 ± 0.8 weeks, *p* = 0.21). Moreover, the physical activities of individual participants during Prehab were recorded weekly.

#### Quadriceps Strength

Maximal quadriceps strength was measured by maximal voluntary isometric contractions (MVIC) using an isokinetic dynamometer (Biodex System 4, Biodex Medical Systems Inc., New York, USA). Before the assessment, participants engaged in a 5-minute warm-up on a stationary bicycle with light resistance. Subsequently, participants were securely positioned with the hip flexed at 90° and the knee flexed at 45° on the seat, using straps over the chest, hip, and thigh to prevent compensatory movements during the assessment. To familiarize themselves with the procedure and warm up the quadriceps, participants performed 3 sub-maximal isometric knee extensions, each held for 5 s. Next, participants were instructed to complete 3 MVICs of knee extension by kicking out as hard and fast as possible with 30 s of rest between each contraction. The highest peak torque among the 3 contractions was collected as the MVIC and normalized by body mass for further analyses.

#### The Capacity for Rapid Contraction in Quadriceps

The capacity for rapid contraction in the quadriceps was assessed through the rate of torque development (RTD), extracted from torque-time curves recorded during the MVIC test. Specifically, early and late RTD were retrieved and calculated as the average slopes of torque versus time curve from 0 to 50ms (RTD_0 − 50_) and 100-200ms (RTD_100 − 200_), respectively. The onset of a contraction was identified as the torque ≥ 20Nm according to a published protocol [27]. The torque data were sampled at 100 Hz. Raw torque signals were filtered using a fourth-order low -pass Butterworth filter with a cutoff frequency of 15 Hz. Data were exported and analyzed using SPSS software (SPSS Version 26). The highest RTD_0 − 50_ and RTD_100 − 200_ were selected and normalized to body mass. The choice of 0-50ms and 100-200ms for RTD was based on distinct physiological determinants during these periods [28], suggesting potential variations in the effects of WBV stimuli on them. Moreover, these intervals play distinct roles in functional performance and knee function among patients with ACL injuries [29, 30].

#### Quadriceps Inhibition

Quadriceps inhibition was quantified by central activation ratio (CAR), measured by the superimposed burst (SIB) technique [31]. CAR is calculated as MVIC/ (MVIC + stimulation provoked torque), and CAR of 1 means complete activation of the quadriceps without any inhibition. Thus, a higher CAR indicates a lower degree of quadriceps inhibition.

Participants were given at least 5 min of rest after finishing the MVIC test to avoid muscle fatigue. The positioning of SIB was identical to that during the MVIC test on the isokinetic dynamometer. Two self-adhesive electrodes [ValuTrode (7.5 × 13 cm), Axelgaard manufacturing, CA, USA] adhered to the quadriceps along the femoral nerve. Participants were then instructed to perform three additional repetitions of 5s extension MVICs with a 1-minute rest interval between each contraction. A supramaximal electrical stimulus (100 pulses/s, 600µs pulse duration, 10 pulses train) was automatically delivered to the quadriceps at the 3rd second during the MVIC by a constant-current electrical stimulator (DS7R; Digitimer, Welwyn Garden City, UK), which was controlled by software (Signal 7.05a, CED Software, Cambridge, UK). The intensity of the supramaximal electrical stimulus was determined before the SIB test. At rest, electrical stimulations were delivered to quadriceps progressively, increasing by 100 mA each time until the torque provoked by the stimulation reached a plateau [32]. To guarantee full stimulation of the quadriceps, 120% plateau current intensity was utilized during the SIB test.

The maximal CAR among 3 successful trials was collected and used for further analyses.

#### Subjective Knee Function

Subjective knee function in the study was measured by the International Knee Documentation Committee (IKDC) Score. Participants completed the IKDC score questionnaire at baseline and preoperatively after finishing the quadriceps assessments. At 4 months postoperatively, the questionnaire was distributed to individual participants by email or mobile phone.

#### Physical Activities

The physical activity level of individual participants was recorded by the International Physical Activity Questionnaire (IPAQ)-Short Form weekly. The IPAQ-Short Form was used to monitor whether participants maintained comparable levels of physical activity outside the intervention during the prehab period, as variations could potentially affect the outcomes. Data collected with IPAQ-Short Form was reported as a continuous measure and was reported as median MET-minutes. Data processing and computation were performed according to the guidelines for data processing and analysis of the IPAQ - Short Form [33].

### Statistical Analysis

Statistical analyses were performed using the SPSS software (SPSS Version 26), following the intention-to-treat principle, and the missing data were filled by the mean value of the group stratified by sex. The normality of quantitative data was checked by the Shapiro-Wilk test. Demographical and baseline data were compared between the two groups using the Independent Student t-test and Chi-square test. However, due to the non-normality of RTD_0 − 50,_ RTD_100 − 200_ in the control group, and RTD_100 − 200_ on the uninjured limb in the WBV group, the Mann-Whitney U test was used for the baseline comparisons of these outcomes. To address the non-normality of some outcome variables and control for potential covariates, linear mixed-effect models were used to compare the changes in quadriceps MVIC, RTD_0 − 50_, RTD_100 − 200_, CAR, and knee function between the 2 groups. Linear mixed-effect models were fit via restricted maximum likelihood, with the group (dummy-coded: 1 = control group, 2 = WBV group), time and their interaction as the fixed effects, subjects as the random effects, time from injury and the baseline assessments of quadriceps MVIC, RTD_0 − 50_, RTD_100 − 200_, CAR, and the IKDC scores as covariates. Multiple linear regression analysis was conducted using a block-wise approach to examine the effects of the changes in the preoperative quadriceps neuromuscular functions measured in this study on the postoperative IKDC scores. In the first block, the changes in quadriceps neuromuscular function from baseline to preoperatively (ΔMVIC, ΔRTD₀₋₅₀, ΔRTD₁₀₀₋₂₀₀, and ΔCAR) were entered to assess the unique contribution of the independent variables to the dependent variable ( the IKDC scores at 4 months postoperatively); in the second block, covariates including intervention group (dummy-coded: 0 = control group, 1 = WBV group), sex, age, BMI, time from injury and the baseline IKDC scores were entered to control for their potential confounding effects. In addition, all assumptions for linear regression were checked, including linearity, homoscedasticity, normality of residuals, and multicollinearity (assessed using variance inflation factor, VIF). All the variables were presented as mean and standard deviation. The statistical significance level was set as *p* < 0.05.

## Results

**Fig. 1:**
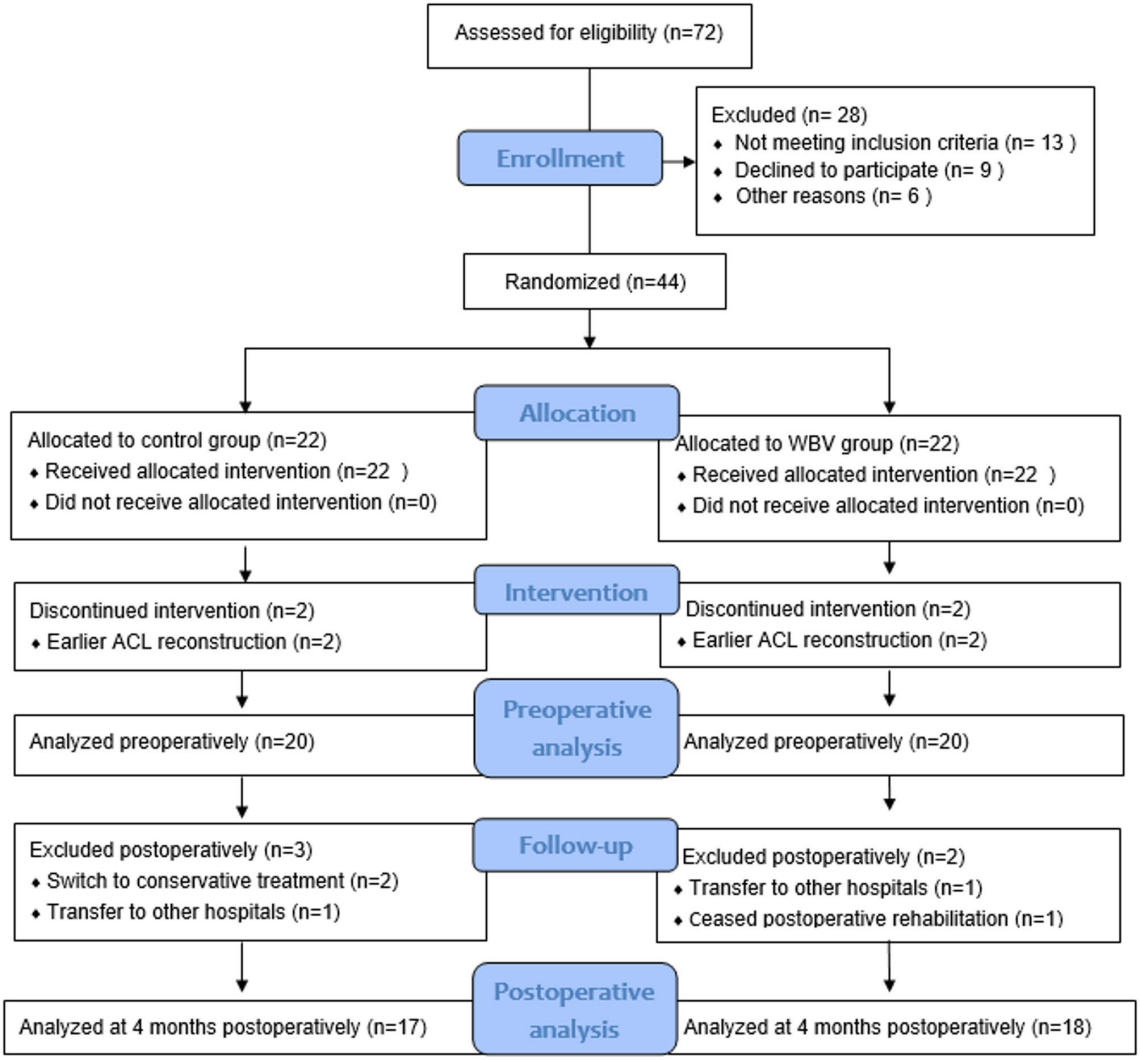
The CONSORT flow diagram displaying the workflow of the study

A total of 72 consecutive patients underwent screening, resulting in the inclusion of 44 participants (28 males and 16 females) with an average age of 27.4 ± 6.7 years, BMI of 23.4 ± 3.0 Kg/m^2^, and pre-injury Tegner score of 7 ± 1. Time from injury to baseline assessment was recorded in weeks, ranging from 2 to 260 weeks. These eligible individuals were then randomized into either the control group (*n* = 22) or the WBV group (*n* = 22). Among the 44 patients, 40 of them completed Prehab and interventions (compliance rate: 91%), 35 finished the postoperative follow-up (compliance rate: 80%), and details can be found in FIGURE 1. Participant demographics, baseline assessments, and physical activities per week during the Prehab and intervention were comparable between the two groups after randomization (see Table 1).

**Table 1:** Demographics and the baseline assessment between the control and the WBV group

	Control group (*N* = 22)	WBV group (*N* = 22)	*P*
Gender (Male, n)	12	16	0.320
Age (years)	26.3 ± 6.2	28.5 ± 7.2	0.277
BMI (Kg/m^2^)	23.1 ± 3.0	23.7 ± 3.0	0.533
Time from injury (weeks)	24.6 ± 61.6	34.4 ± 55.2	0.581
Pre-injury Tegner score	7 ± 1	7 ± 1	0.277
Meniscal injuries (n)	7	8	0.750
IKDC score	59.8 ± 10.2	65.9 ± 14.6	0.115
Injured MVIC (Nm/Kg)	1.65 ± 0.38	1.81 ± 0.45	0.215
Injured RTD_0 − 50_(Nm/Kg*s)	6.00 ± 3.85	7.86 ± 4.97	0.201
Injured RTD_100 − 200_(Nm/Kg*s)	2.64 ± 1.54	2.96 ± 1.62	0.630
Injured CAR	0.925 ± 0.043	0.946 ± 0.032	0.078
IPAQ Score (MET*min)	2405 ± 1613	2256 ± 1438	0.718

The results are presented as mean ± standard deviation

BMI: body mass index, IKDC: International Knee Documentation Committee, MVIC: maximal voluntary isometric contraction, RTD: rate of torque development, CAR: central activation ratio, IPAQ: International Physical Activity Questionnaire

### The Effects of WBV on Quadriceps Strength from Baseline To Preoperatively

**Fig. 2:**
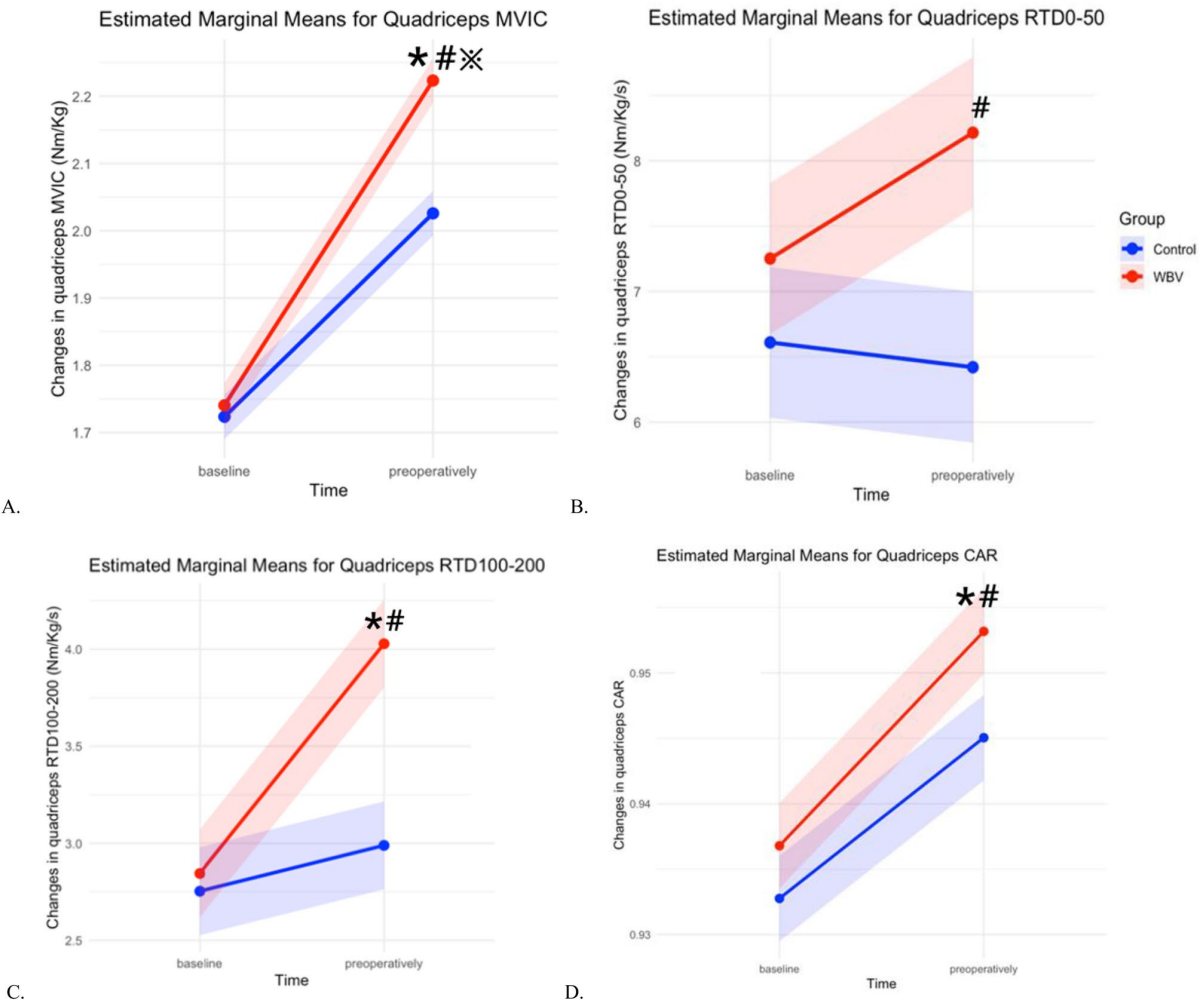
The changes of quadriceps neuromuscular function from baseline to preoperatively The dots represent the mean of each variable, while the dashed areas indicate the residuals of the estimated marginal means. Abbreviations: MVIC = maximal voluntary isometric contraction; RTD = rate of torque development; CAR = central activation ratio; *indicates the significant main effect of time on the outcome; #indicates the significant main effect of intervention on the outcome; ※indicates the significant time*intervention interaction on the outcome; significance was set at *p* < 0.05

After adjusting for baseline quadriceps MVIC and time from injury, time (F = 133.73, *p* < 0.001), intervention (F = 10.790, *p* = 0.002), and the interaction of time and intervention (F = 7.067, *p* = 0.011) showed significant main effects on quadriceps MVIC in the injured limb from baseline to preoperatively. The quadriceps MVIC increased 0.39Nm/Kg on average (95%CI: 0.32, 0.46). The WBV group showed greater improvements compared to the control group, with an average difference of 0.107 Nm/Kg (95% CI: 0.041, 0.173) (see FIGURE 2A).

### The Effects of WBV on the Capacity of Rapid Contraction in the Quadriceps from Baseline To Preoperatively

From baseline to preoperative assessment, after adjusting for baseline RTD_0 − 50_ and time from injury, the intervention showed a significant main effect on quadriceps RTD_0 − 50_ (F = 5.512, *p* = 0.024), with an average improvement of 2.35 Nm/Kg/s (95%CI: 0.40, 4.38). However, neither time (F = 0.351, *p* = 0.557) nor the time *intervention interaction (F = 0.779, *p* = 0.382) demonstrated significant main effects on quadriceps RTD_0 − 50_ (see FIGURE 2B). For quadriceps RTD_100 − 200_, both time (F = 7.650, *p* = 0.008) and intervention (F = 8.878, *p* = 0.005) exhibited significant main effects after adjusting for baseline RTD_100 − 200_ and time from injury, while time*intervention interaction was not significant (F = 3.409, *p* = 0.072). From baseline to preoperatively, RTD_100 − 200_ improved by an average of 0.710Nm/Kg/s (95%CI: 0.192, 1.227). Although the WBV group showed numerically greater improvements compared to the control group (mean difference: 0.566 Nm/kg/s, 95% CI: 0.182, 0.948), the time * intervention interaction was not statistically significant (*p* = 0.072), indicating that the between-group difference in improvement over time was not supported statistically (see FIGURE 2C).

### The Effects of WBV on Quadriceps Inhibition from Baseline To Preoperatively

After adjusting for time from injury and the baseline CAR, both time (F = 15.852, *p* < 0.001) and intervention (F = 4.352, *p* = 0.043) demonstrated significant main effects on quadriceps CAR, with the WBV group had greater improvements of 0.006 (95%CI: 0.001, 0.012). However, the interaction between time and intervention was insignificant (F = 0.324, *p* = 0.573). (see FIGURE 2D).

### The Effects of WBV on Subjective Knee Function from Baseline To 4 Months Postoperatively

**Fig. 3:**
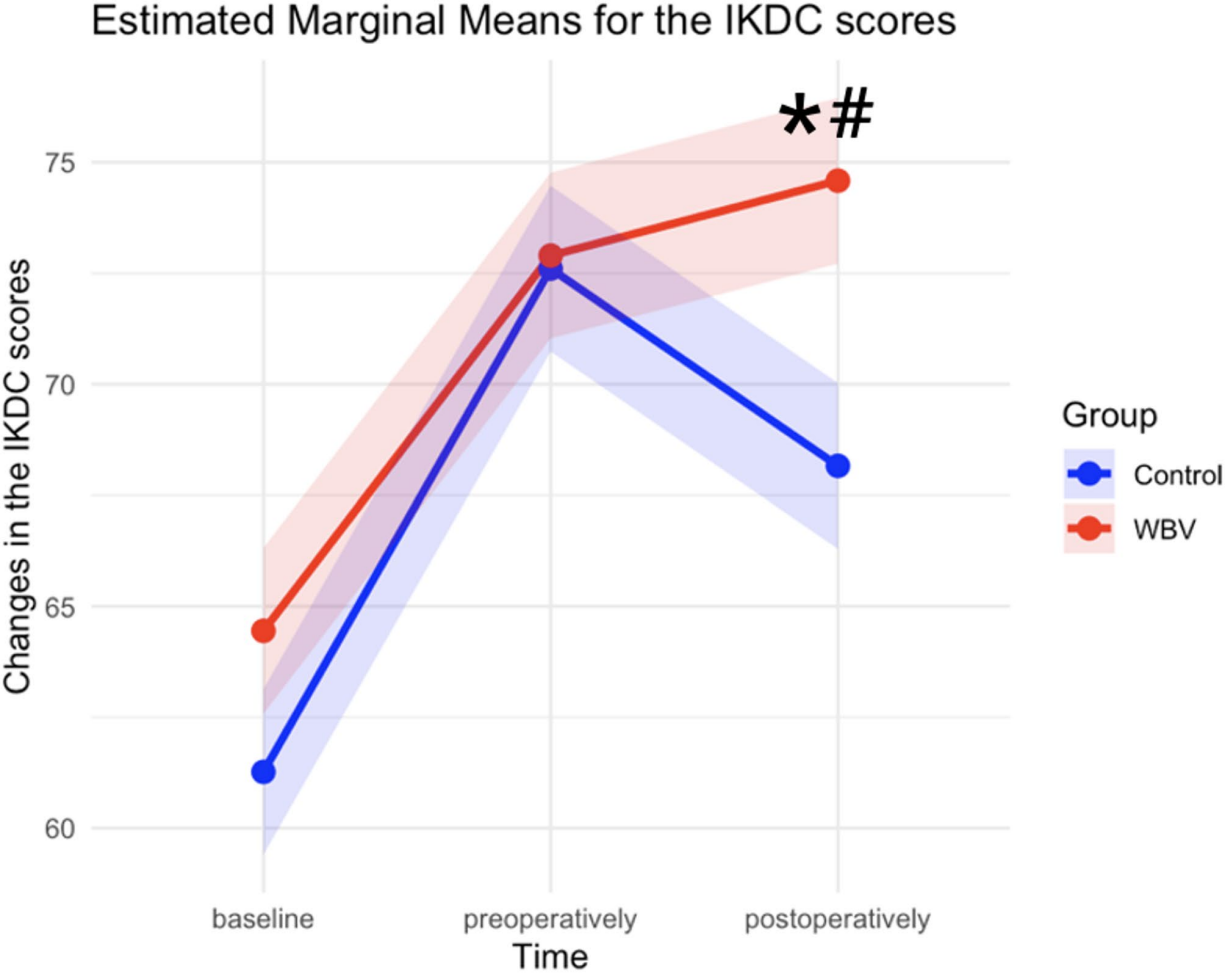
The IKDC score from baseline to 4 months postoperatively The dots represent the mean of each variable, while the dashed areas indicate the residuals of the estimated marginal means. *indicates the significant main effect of time on the outcome; #indicates the significant main effect of intervention on the outcome; significance was set at *p* < 0.05

After adjusting for time from injury and the baseline IKDC scores, both time (F = 14.587, *p* < 0.001) and intervention (F = 8.288, *p* = 0.006) had significant main effects on the IKDC score. The IKDC score improved significantly from baseline to preoperatively and 4 months postoperatively, with average improvements of 9.89 (95%CI: 5.05, 14.74) and 8.52 (95% CI: 3.67, 13.36), respectively. However, there were no significant differences in the IKDC sores between preoperatively and 4 months postoperatively (*p* = 0.549). Although the time * intervention interaction was not statistically significant (F = 1.195, *p* = 0.308), indicating no differential change over time between groups, the WBV group had a higher IKDC score compared to the control group, with a mean difference of 5.60 (95% CI: 1.67 to 9.53). (see FIGURE 3).

### The Effects of the Changes in Preoperative Quadriceps Neuromuscular Function on Postoperative Knee Function

The stepwise multiple linear regression analysis revealed that both the ΔMVIC and intervention were predictors of postoperative IKDC scores. In the first model, ΔMVIC was identified as a significant predictor, accounting for 9% of the variance in the IKDC scores post-ACLR (R^2^ = 0.090, ΔR²=0.068, F = 4.145, *p* = 0.048). In the second model, the addition of the intervention variable significantly improved the model fit, increasing the explained variance to 23.9% (R^2^ = 0.239, ΔR²=0.149, F = 6.433, *p* = 0.007). These findings highlight the combined influence of quadriceps strength recovery from baseline to preoperatively and the intervention on postoperative IKDC scores. The details can be found in Table 2.

**Table 2:** The results of the multiple linear regression analysis

Model	Variables	Unstandardized β	Standardized coefficients β	t value	*P* value	VIF
1	Constant	66.452		23.515	< 0.001	
	ΔMVIC	12.537	0.300	2.036	0.048	1.000
2	Constant	56.602		13.011	< 0.001	
	ΔMVIC	5.913	0.141	0.960	0.343	1.168
	Intervention	8.299	0.417	2.833	0.007	1.168

Dependent variable: The IKDC scores 4 months postoperatively

## Discussion

The results of this study were consistent with our hypothesis. Our study revealed that the Prehab incorporating WBV demonstrated potential to improve knee function following ACLR. Furthermore, as hypothesized, we found that the enhancement in preoperative quadriceps strength associated with better knee function following ACLR.

To the best of our knowledge, the application of WBV in Prehab for ACLR has not been reported previously. Similar to earlier studies that demonstrated improved quadriceps strength by incorporating WBV into postoperative rehabilitation of ACLR [22], our study, which focuses on Prehab, also showed comparable effects of WBV on increasing quadriceps strength before ACLR. In addition, the physical activity levels outside of the intervention, as measured by the IPAQ, were similar between groups throughout the prehabilitation period, indicating that differences in outcomes were not likely attributable to differences in overall physical activity. One strength of our study is that, unlike previous studies merely adding a WBV exercise program along with conventional rehabilitation, our control group performed identical squat exercises without WBV stimuli. This study design allowed for a focused exploration of the specific effects stemming from the WBV [22].

Research exploring the effects of WBV on rapid quadriceps contraction, particularly in patients with ACL deficiency, remains limited [22]. In our study, after adjusting the baseline value and time from injury, the intervention demonstrated a significant main effect on quadriceps RTD_0 − 50_, indicating that the WBV had a potential superior effect on enhancing quadriceps RTD_0 − 50_. However, neither time nor the time * intervention interaction reached statistical significance from baseline to the preoperative assessment. These results indicate that while WBV was associated with greater overall improvements in RTD_0 − 50_, it did not lead to a significantly different RTD_0 − 50_ compared to control group. Given that RTD_0 − 50_ is primarily influenced by the excitability of motor neurons in muscles, our finding suggests that our WBV program might have positive effects on modulating motor neuron excitability in the quadriceps. This interpretation is further supported by the significant effects of WBV on quadriceps CAR (see FIGURE 2D) which is also predominantly determined by motor neuron excitability. The lack of time-dependent effects may reflect the relatively short duration or limited intensity of the intervention, which may have been insufficient to produce more pronounced temporal changes.

Our study found that both time and intervention had significant main effects on quadriceps RTD_100 − 200_ from baseline to preoperatively, indicating that RTD_100 − 200_ improved over time and that WBV therapy was associated with overall greater improvements across participants. The time * intervention interaction did not achieve the significant level (*p* = 0.072), suggesting the trajectory of improvment did not differ statistically between groups. The nonsignificant interaction effect may reflect insufficient statistical power due to sample size or limited WBV dosage or duration to detect group differences in trajectories of change between groups. Unlike RTD_0 − 50_ which is more dependent on neural drive, RTD_100 − 200_ is primarily determined by the contractile properties of the muscle [15]. Previous research has shown that WBV can down-regulate negative muscle regulators such as myostatin and atrogin-1, while simultaneously increasing levels of anabolic hormones such as testosterone and growth hormone [34, 35]. These mechanisms may help explain the observed improvements in RTD₁₀₀–₂₀₀ following the 5-week WBV program.

Our study found significant main effects of both time and intervention on quadriceps CAR, suggesting that WBV was associated with more alleviation of quadriceps inhibition in the participants. However, the absence of a significant interaction effect between time and intervention indicates that the changes of quadriceps CAR from baseline to preoperatively did not reach a significantly different level between the 2 groups. These findings indicate that the volume or duration of WBV therapy employed in this study—or the relatively limited sample size—may have been insufficient to produce a statistically significant difference in the rate of improvement between groups. Pamukoff et al. reported that WBV could immediately increase quadriceps CAR by enhancing corticospinal excitability in patients years after ACLR [21]. However, Colson et al., using a similar WBV protocol, observed no significant changes in quadriceps inhibition among physically active college students when comparing WBV and sham groups [36]. The different findings may be attributed to varing levels of baseline quadriceps inhibition across studies. Notably, patients with ACLR tend to exhibit more pronounced quadriceps inhibition, which could explain the contrasting findings. In our study, participants’ quadriceps inhibition prior to ACLR may have been less severe, potentially reducing the detectable impact of WBV. Future studies with larger samples and more intensive or prolonged WBV protocols are warranted to better evaluate the efficacy of WBV prehabilitation on quadriceps neuromuscular activation.

Evidence shows that preoperative quadriceps strength and the intervention are positively associated with knee function following ACLR [6]. In this study, incorporating WBV into Prehab significantly increased quadriceps strength prior to ACLR. Based on our results, the additional improvements in quadriceps strength observed in the WBV group likely contributed to higher IKDC scores post-ACLR. These findings highlight the critical role of preoperative quadriceps strength and WBV therapy in improving knee function outcomes after ACLR. Our results are consistent with the findings of Hartigan et al. [37].

It is important to note that while we observed statistically significant difference in IKDC scores from baseline to 4 months post-ACLR between the control and the WBV group, the magnitude of the difference 5.60 falls below the minimally clinically important difference (MCID) threshold of approximately 19 points suggested by Beletsky et al. for ACLR patients [38]. Similarly, the observed greater improvements in quadriceps MVIC in the WBV group did not achieve the MCID (3Nm/Kg) of quadriceps strength in individuals with ACLR [39]. However, these improvements should be considered within the context of the Prehab timeframe. The 5-week intervention represents only the beginning of the treatment continuum, and these early improvements, while modest, may create a favorable foundation for continued recovery throughout the lengthy postoperative rehabilitation process. Future studies with longer follow-up periods are needed to determine if these early advantages translate to clinically meaningful differences in long-term outcomes.

To the best of our knowledge, this study pioneers the use of WBV in the preoperative phase for ACLR patients, considering a comparable control group and the interactive effects of WBV with other exercises in the Prehab program. However, this study still has some limitations. Firstly, the standard WBV protocol has not been established; thus, the present study can only demonstrate the effects of the current 5 weeks of WBV protocol on the outcomes. Secondly, we only followed up the participants to 4 months postoperatively. A long-term follow-up is necessary to evaluate whether the benefits of WBV in Prehab can be sustained throughout the entire postoperative rehabilitation process. Thirdly, quadriceps neuromuscular function was not assessed at 4 months postoperatively due to practical constraints. Many participants were unable to attend in-person testing because they had returned to work or faced geographical limitations. Future studies should investigate whether preoperative WBV therapy can lead to improved quadriceps neuromuscular function post-ACLR. Fourthly, our study included only patients undergoing ACLR with hamstring autografts, so the results cannot be extended to those receiving bone-patellar tendon-bone grafts or other graft types, which may influence quadriceps function differently during Prehab. Then, we only measured quadriceps neuromuscular function at a single position at knee flexion at 45°. This position was chosen because its significant association with knee function [32]. However, the open-kenetic chain testing position was different from real-life tasks in daily life, which may limit the generalizebility of the findings. Additionally, the use of a 100 Hz sampling rate for torque data, which may have underestimated RTD_0 − 50_ and introduced potential aliasing errors. This constraint, due to equipment limitations, may have affected the absolute accuracy of RTD_0 − 50_, although relative group comparisons remain interpretable due to standardized protocols. Finally, based on the current findings, it appears that the sample size or the volume of WBV used in this study may have been insufficient to elicit a significantly greater rate of improvements in quadriceps neuromuscular function and knee function. Future studies with larger sample sizes and higher WBV volumes are warranted to further explore its potential benefits.

## Conclusions

In conclusion, the 5-week WBV in Prehab had potentials to improve knee function after ACLR. In addition, the improvements in preoperative quadriceps strength contributed to improved knee function post-ACLR.

## Supplementary Information

Below is the link to the electronic supplementary material.

Supplementary Material 1

## Data Availability

The datasets generated and/or analysed during the current study are not publicly available due to the protection of our participants’ privacy but are available from the corresponding author on reasonable request.

## Abbreviations

ACL: Anterior cruciate ligament
ACLR: Anterior cruciate ligament reconstruction
WBV: Whole-body vibration
Prehab: Prehabilitation
IKDC score: International Knee Documentation Committee Score
MVIC: Maximal voluntary isometric contraction
IPAQ: International Physical Activity Questionnaire
RTD_0 − 50_: Rate of torque development from 0 to 50ms
RTD_100 − 200_: Rate of torque development from 100 to 200ms
CAR: Central activation ratio
SIB: Superimposed burst technique
