# Supplementary material for: Five Weeks of Whole-body Vibration in Prehabilitation for Knee Function Following Anterior Cruciate Ligament Reconstruction: A Single-blinded Randomized Controlled Trial
Source: Sports Med Open. 2025 Aug 22;11:98. doi: 10.1186/s40798-025-00901-1 (PMC12373618; doi:10.1186/s40798-025-00901-1)
Supplement: Supplementary file 1 — Supplementary Material 1 [file 40798_2025_901_MOESM1_ESM.pdf]

Journal: Sports Medicine-Open

Title: Five weeks of whole-body vibration in prehabilitation for knee function following anterior cruciate ligament reconstruction: A single-blinded randomized controlled trial

Authors: Jihong Qiu[1], PhD; Michael Tim-Yun Ong[2], FRCS Ed (Orth); Chi-Yin Choi[2], MSc; Mingde Cao[2], PhD; Violet Man-Chi Ko[2], PhD, PT; Xin He[2], PhD; Sai-Chuen Fu[2], PhD; Daniel T.P. Fong[3], PhD; Patrick Shu-Hang Yung[2*], FRCS Ed (Orth)

Affiliations: 1. Shanghai University of Sport, School of Exercise and Health, Shanghai, China

2. Department of Orthopaedics and Traumatology, Faculty of Medicine, The Chinese University of Hong Kong, Hong Kong SAR, China

3. National Centre for Sport and Exercise Medicine, School of Sport, Exercise and Health Sciences, Loughborough University, Loughborough, LE11 3TU, UK

Supplementary File 1. Descriptions of Prehab Program

Table 1. The descriptions of Prehab program according to Exercise Reporting Template (CERT)

Item Category	Item No.	Descriptions of the Prehab Program
What: Materials	1	· Monark Bicycle<br>· Wobbles cushion<br>· Bosu Ball<br>· Leg extension machine<br>· Leg curl machine<br>· Resistance Thera-band<br>· Kettle-bell
Who: Provider	2	The first and the second author of this study, who obtained the qualifications of athletic training or physical therapy.
How: Delivery	3	The exercises were performed individually.
	4	The exercises were supervised.
	5	The research team recorded and measured the adherence to exercise of every participant. All the participants must complete all the 10 sessions of Prehab before ACLR.
	6	To enhance participant motivation, we emphasized the importance of Prehab on the outcomes following ACLR to all the participants and promised to deliver a comprehensive assessment report to their surgeon for clinical reference.
	7	Regular evaluations of exercise progression occurred on a weekly basis, taking into account the clinical condition of each participant. Adjustments to the strengthening exercise intensity were made in accordance with the progression models recommended by the American College of Sports Medicine.
	8	The description of each exercise can be found in Supplementary File 1(Table 2).
	9	Theoretically, the Prehab program did not include any home-based component. However, to minimize the potential confounding from daily activities, we monitored physical activities of each participant weekly by IPAQ.
	10	Cryotherapy was delivered to the participants at the end of each session to avoid repeated knee swelling.
	11	If the participant complained pain or persistently

		repeated swelling happed to the knee joint, we recorded the status and suspended the exercise immediately.
Where: location	12	The Prehab program was conducted in a sports biomechanics and performance lab.
When, How much: dosage	13	The description of each exercise can be found in Supplementary File 1. Table 2.
Tailoring: what, how	14	All the participants took the standard Prehab program in this study. However, the intensity of each component in the program was tailored to each participant, which is described at item 15.
	15	The starting level for proprioception training was based on the participant's perceived difficulty, with exercises initiated at a moderate difficulty level as self-reported. For resistance training, the starting load was determined using an estimated one-repetition maximum (1RM), calculated via the Brzycki formula based on the participant's performance to momentary muscular failure within the 8–12 repetition range during their initial visit. The starting level for functional training was guided by the participant's clinical status and perceived exertion using the Borg CR-10 scale; exercises were initiated and maintained at a level corresponding to a score of 5-7 out of 10.
How well: planned, actual	16	The Prehab program was delivered and performed as planned.

Table 2. Prehabilitation protocol

**Preoperative rehabilitation protocol (twice/week, five weeks)**			
	**Types**	**Intensity**	**Progression**
**Warm up**	Cycling	5 min with moderate resistance	NA
**Balancing and proprioceptive exercises**	Stand on a Wobbles cushion	30 s * 3 sets	1. Standing on double legs with eyes closed<br>2. Standing on single leg with eyes open<br>3. Standing on single leg with eyes closed
	Squat on a Bosu ball	10 reps * 3 sets	Squats with increased speeds
**Strengthening exercises**	Load bearing squats	75% RM* 12 reps *3 sets	The 1 repetition maximum was estimated every week, and the loading was adjusted accordingly.
	Leg extension	75% RM* 12 reps *3 sets	
	Leg curl	75% RM* 12 reps *3 sets	
	Hip abduction	75% RM* 12 reps *3 sets	
**Functional training**	Jogging	200 meters	NA
	Deep jump with soft landing	10 reps * 2 sets	
	Single leg hop (forward, lateral, cross-over)	10 reps * 2 sets	
